# Hospital treatment costs associated with incident complications in patients with type 2 diabetes—real-world study based on electronic patient information systems

**DOI:** 10.1186/s12913-022-07895-6

**Published:** 2022-04-09

**Authors:** Reijo Sund, Tuomas Peltonen, Aku-Ville Lehtimäki, Janne Martikainen

**Affiliations:** 1 Institute of Clinical Medicine, School of Medicine, University of Eastern Finland, PO Box 1627, 70211 Kuopio, Finland; 2 Medical Affairs, MSD Finland, Espoo, Finland; 3 School of Pharmacy, University of Eastern Finland, Kuopio, Finland

**Keywords:** Type 2 diabetes, Costs, Real-world data, Complications

## Abstract

**Background:**

Type 2 diabetes (T2D) and its complications cause a significant public health and economic challenge. To enable the optimal resource allocation across different prevention and treatment policies for the management of T2D-related complications, detailed cost estimates related to the complications of T2D are needed. Therefore, the objective of the study was to provide reliable and sufficiently detailed real-world estimates of costs associated with different T2D complications in a Finnish university hospital setting.

**Methods:**

A cohort of T2D patients living in the catchment area of a university hospital during 2012 and 2016 was identified from the comprehensive national FinDM diabetes database for longitudinal assessment of T2D associated complication treatment costs. Data on patient-level events were extracted from the FinDM data and complemented with all accountable services and related detailed costing data gathered from the university hospital’s electronic patient information systems by using unique personal identity codes. Patients were screened for their first diagnoses of complications using the same national quality registry definitions as in the FinDM database. Multivariable gamma regression model with a log link function was applied to study the association between baseline factors and complication costs. In addition, an interactive online tool was developed to create predicted costs for complication costs with selected baseline factors.

**Results:**

A total of 27 255 prevalent and incident patients with T2D were identified from the national FinDM register. Finally, a total of 16 148 complication episodes for 7 895 patients were included in the cost analyses. The mean estimated one-year hospital treatment costs of T2D-related complication varied from 6 184 to 24 507 euros per complication. Regression analyses showed that coexisting conditions are significantly associated with initial and recurrent complication costs.

**Conclusions:**

The study shows updated Finnish cost estimates and their main cost drivers for T2D-related complications treated in the university hospital setting. The results of our study highlight the significance of guideline implementation, effective preventive treatments for T2D, as well as the importance of treatment adherence to avoid these costly complications.

**Supplementary Information:**

The online version contains supplementary material available at 10.1186/s12913-022-07895-6.

## Background

The increasing epidemic of type 2 diabetes (T2D) and its complications cause a significant public health and economic challenge. Currently, the global prevalence of T2D is around 8,4% [1]. T2D is a manifestation of genetic, environmental, and lifestyle-associated factors, such as sedentary lifestyle, energy-dense diets, and population ageing [1, 2]. T2D is a metabolic disorder in which chronic high levels of blood glucose result from inability of insulin to act on cells due to insulin resistance [1], sometimes combined with an insufficient amount of insulin production [2].

It is not only the disease itself but mainly complications, that adversely affects to quality of life, life expectancy and being behind the overall costs around to T2D [1]. Complications have traditionally been divided to microvascular (i.e., neuropathy, retinopathy, and nephropathy, respectively) and macrovascular complications (i.e., hypertension, myocardial infarction (MI), congestive heart failure, stroke, and peripheral artery disease) [3]. In Finland, where the prevalence of T2D is currently around 15% among males and 10% among females [4], diabetes in general causes annually on average 833 M€ excess health care costs (in year 2011 value), which is around 9% of total annual health care costs. Unfortunately, these current national aggregate-level cost estimates do not provide detailed information on the costs of individual micro- and macrovascular complications or the influence of sociodemographic or clinical determinants on these complication costs. These type of detailed cost estimates are needed to support policymakers and health care providers in evaluating the optimal resource allocation across different prevention and treatment policies for the management of T2D-related complications. In addition, detailed cost estimates are also needed when evaluating the cost-effectiveness of novel T2D treatments aiming to lower the high levels of blood glucose and other risk factors associated with the incidence of T2D-related complications in different patient populations as a part of the national pricing and reimbursement (P&R) processes of medicines, such as the P&R process maintained by the Pharmaceutical Pricing Board in Finland. Therefore, the aim of the present study was to provide reliable and sufficiently detailed real-world cost estimates and their determinants associated with different T2D complications in Finland.

## Methods

### Study setting

In Finland, patients with T2D are typically treated and monitored in primary health care, where over 300 municipalities (local authorities) are responsible for the provision of these services to their residents. Health centres commonly have General Practitioner (GP)-run inpatient units, largely for chronic and long-term care patients. In addition, these Finnish municipalities form 20 hospital districts to provide hospital care as secondary and tertiary settings. Secondary care (including specialised outpatient care, inpatient care, and day surgery) is mainly provided by hospitals and tertiary care is delivered in five university hospitals. Patients need a referral to access specialist care, except for emergency cases. The management and follow-up of T2D patient population with micro- and macrovascular complications takes place in hospital setting in Finland.

The present study was conducted in Kuopio University Hospital (KUH), which provides the secondary health care services for around 250 000 citizens in its catchment area. In addition, it provides highly specialised tertiary health care services for around 810 000 inhabitants in Eastern and Central Finland, which is around 15% of all Finnish citizens (*N* = 5.5 million).

### Study population and data linking

In the present study, people with diabetes were identified from the comprehensive national FinDM diabetes database [5]. The national FinDM database includes all Finnish patients with diabetes between years 1964–2017 and their linked health care data from several national registers. For the purposes of the present study, the T2D population having a home municipality code (any time during 2012–2017) within the catchment area of KUH (the Hospital District of Northern Savo) or any treatment at KUH during 2012–2017 were identified from the FinDM database following its definition for T2D [5]. For these patients, detailed data on patient-level events (of which only minor part were directly available from the FinDM database) including inpatient days, surgical procedures, ED visits, physician and nurse outpatient visits, laboratory examinations, diagnoses, medications (utilized during hospitalizations) as well as other accountable services and related detailed hospital claim data were gathered from KUH’s information systems for the period 2012–2018 by using unique national personal identity codes.

**Fig. 1:**
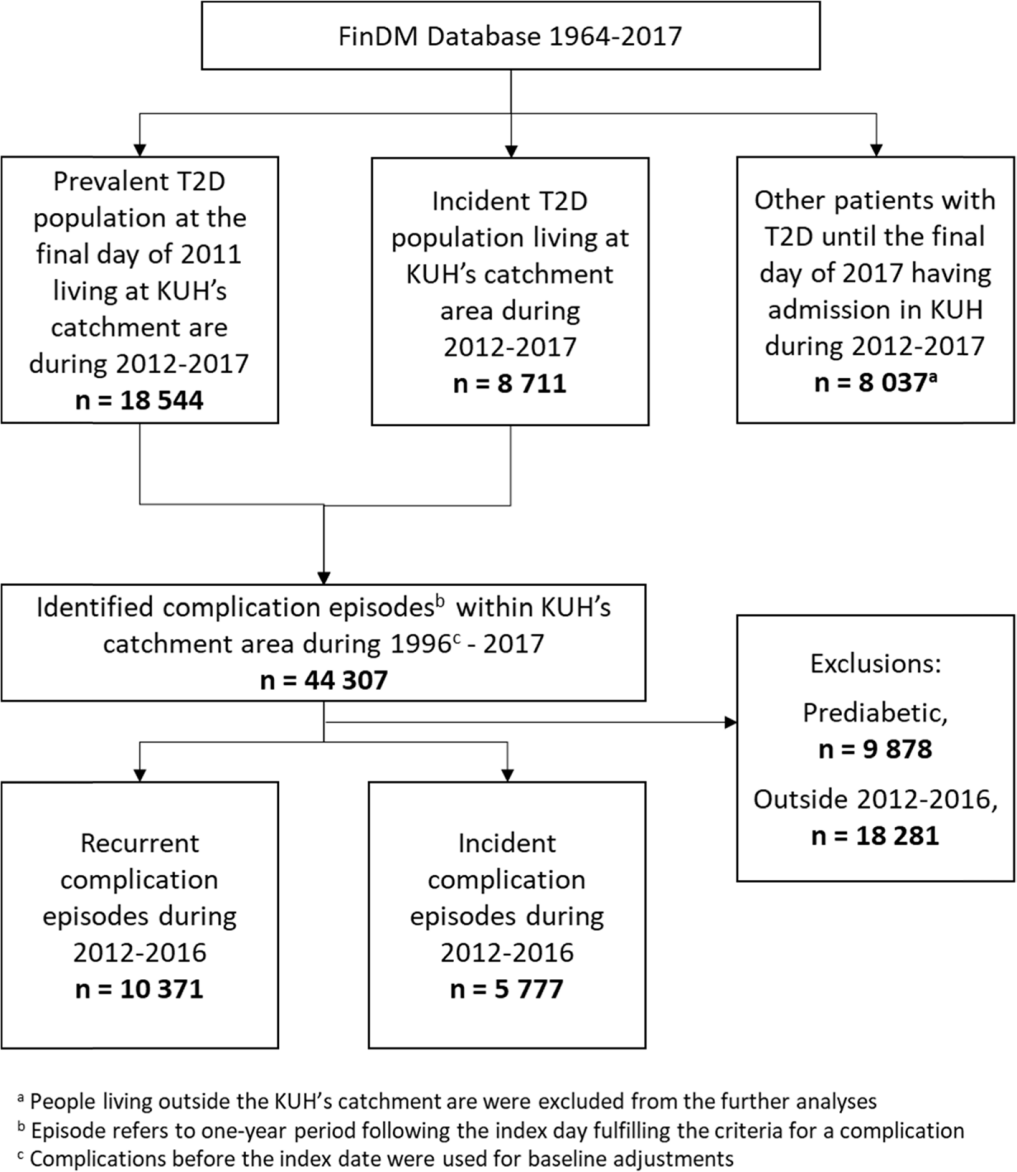
Flow chart describing the selection of patients with T2D and their complication episodes in the present study

From this population, prevalent cases were defined as patients with T2D living in KUH’s catchment area at the final day of December 2011, whereas incident cases were defined as patients with newly diagnosed T2D between years 2012–2017. People living outside the KUH’s catchment area were excluded from the analyses. There were totally 35 292 individuals to whom the linkage to the KUH data was conducted. Of these, 27 255 were finally included to the actual study cohort (Fig. 1).

Next, the complications of these patients were screened during the years 1996–2017 using the same national quality register definitions as in FinDM [5], i.e., by identifying the given ICD-10 diagnoses or operation codes from the hospital admissions for the cerebrovascular, neurological, foot, nephropathy, eye, and cardiovascular complications (Supplement Table 3). The first diagnosis was selected as an index event of complication and all repeated diagnoses of the same complication class within a one year since the index event were considered to belong to the same “episode”, i.e. the diagnosis was considered as a new complication event if it occurred at least one-year since the previous complication event. In other words, the complication “episode” corresponds to the one-year period since the index event fulfilling the criteria of a complication.

Look back-period of ≥ 15 years allowed to further classify the episodes to prediabetic, incident or recurrent episodes depending on the timing of the episode, and diabetic complication episodes with a start date at the KUH during the years 2012–2016 were included in the main analyses to guarantee complete data for the one-year follow-up period (Fig. 1). Comorbid diseases were detected for each episode using disease classes based on the Charlson comorbidity classes [6] by screening for the hospital diagnoses within two years preceding the episode. Due to small number of cases, classes of HIV/AIDS and hemiplegia/paraplegia were dropped from the analyses, and liver diseases as well as malignancies being originally categories with two classes were reduced to any chronic liver disease and any malignancy. In addition, diabetes without complications was dropped as all patients had T2D. Diabetes with complications was kept in the analyses as its definition was based on the recent type of T2D diagnosis and not on the actual diagnoses of complication disorders.

### Resource use and unit costs

Resource use and costs were considered from the health care payer perspective excluding patients’ out-of-pocket costs, direct non-health care costs such as travelling, and productivity loss costs due to T2D-related complications. Thus, health care resource use and costs considered in this study included all inpatient days, surgical procedures, emergency department visits, physician and nurse outpatient visits, laboratory examinations, diagnoses, medications (utilized during hospitalizations) as well as other accountable services related the treatment and monitoring of patients with T2D complications.

To evaluate costs at the complication level from the payer’s perspective, the costs taking place at more general resource related aggregate-level (e.g. salaries of the staff, purchases of equipment and drugs needed for the treatment, maintenance costs of operating rooms and other required facilities etc.) must be assigned to actual outputs, i.e. to the treatment of patients at the hospital. The standard way is to assign the costs to the observed components of the given treatment using suitable weights determined using e.g. diagnosis related group (DRG) or per diem costs. In the current study, the unit and total costs were obtained from the detailed patient-specific municipality billing data linked to the study dataset from KUH’s financial administration system. The KUH’s financial administration data provides an exceptionally detailed classification of the components of the treatment that allows comprehensive “reconstruction” of the actual treatment costs. Those costs are linked to the primary admission (inpatient period or outpatient visit) and the calculated cost is the price that is accounted from the payer of the treatment (a municipality of the patient).

As part of the treatment during the follow-up period have been given in the other (local) hospitals within the hospital district, costs for other hospital care than given in KUH were calculated using the CRHC data containing reported costs for part of the service providers, and for the rest of the costs of in- and outpatient admissions observed in the CRHC data were approximated by weighting the available treatment data (DRG-group or specialty-specific treatment days) using the published unit prices of health care [7].

Finally, we had component specific cost data for each admission (costs time stamped to accounting/discharge date). All costs were deflated to the price level of 2019 using a price index for health care. We used those to derive the cost outcomes of which the one-year costs after the complication event was the primary one. One-year costs are widely used in health economics and capture the burden of costs associated with diabetic complications in a way that also allows comparisons to any other cost-estimates of one-year hospital costs. As the KUH is a public non-profit university hospital with accurate costing system operating in the Finnish welfare state providing universal access to care, it is likely that the costs mapped to the components reflect the “real” costs of the treatment in a generalizable way – at least in the sense that costs within the hospital are on the same scale so that the relative differences are reliably reflecting the real differences of costs within hospital.

### Statistical analysis

The statistical analysis unit was a complication event and there were six main classes of complications. For each complication event, three observation time periods were used: 1) the first year since the onset of complication, 2) the year preceding the complication, and 3) the second year since the onset of complication. The later time period was expected to reflect the ongoing long-term costs of complications, such as rehabilitation and monitoring, but also subsequent events of the same type [8, 9]. The complication events were further divided to first (incident) ones and to recurrent ones so that also the potential effect of repeated complications could be detected. Costs were stratified by detailed hospital resource categories. Cross tabulations, means and standard deviations as well as visualisations using the bar charts and radar plots were used for the descriptive analyses.

In addition, multivariable gamma regression models with log link functions taking an account to the positive skewness of cost data were applied to study the effects of patient characteristics on one-year costs and to generate covariate-adjusted mean estimates of one-year costs. For the incident complications, studied variables included sex, age, duration of T2D, and several comorbidities. In addition, death during the period of one year after the complication was included as a variable as that is known to modify the costs of treatment [10]. For the recurrent complications two additional variables were used: the number of earlier episodes of the same type and an indicator for exceptionally high (> 50 000€) one-year cost of the previous complication episode. These high costs represent a static construction of a variable that would adequately capture 10% of highest costs by using the 90% percentile as a cut point. This cut point approximately corresponded to the limit of 2 SD giving a further justification for the cut point. Regression models were estimated for each incident and recurrent complication type independently (i.e., 12 models in total). Sandwich estimator R package [11, 12] was used to consider the potential effect of clustering, due to the possible multiple recurrent complications of the same type on a single patient. In addition, average marginal effects of regression coefficients were estimated using Margins R package [13].

To dynamically estimate cost distributions for the patients with varying background characteristics, a Cholesky decomposition approach based on regression coefficients and covariance matrices of the models was used to simulate a large amount of possible predictions of the model for the populations with wanted background characteristics. In addition, freely accessible website application was developed for the further analysis of complication costs and their modifiers (https://uef-phoru.shinyapps.io/T2DCost/). The developed website application fits, in real time, customised regression models based on user-selected subset of predictors to predict the hospital treatment costs of different T2D-related complications.

Software package R version 4.0.2 was used for data processing and statistical analysis. Dynamic simulations utilize the Shiny application to run interactive R code.

## Results

### Sample characteristics

**Fig. 2:**
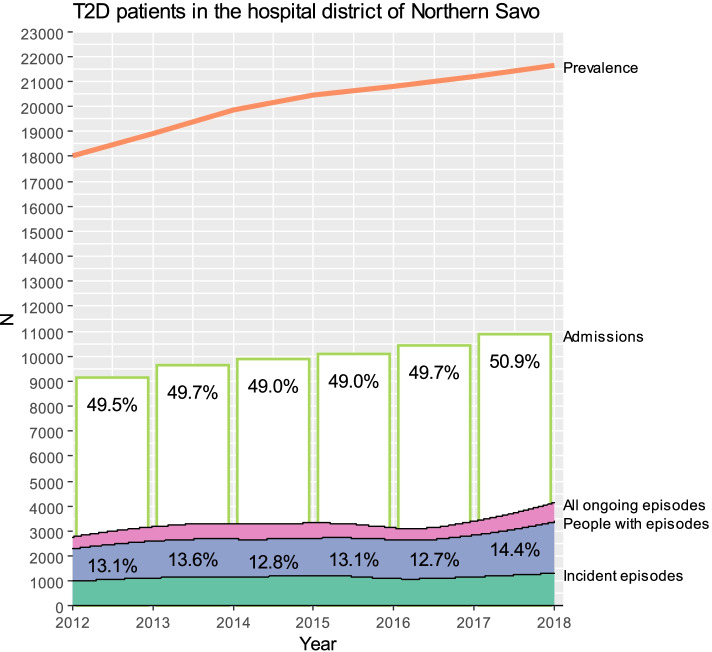
People with T2D in the Northern Savo area. Prevalence of T2D, people with T2D having at least one admission during the year at Kuopio University Hospital (percentages at bars), Number of ongoing T2D complication episodes (i.e. the number of cases with a complication event less than year ago), Number of individuals with at least one ongoing T2D complication episode (percentages), Number of ongoing incident T2D complication episodes (i.e. having the first complication event less than one-year earlier)

A total of 27 255 prevalent and incident patients with T2D living in the hospital district of Northern Savo during the years 2012–2017 were identified from the national FinDM register. Prevalence of T2D at the hospital district of Northern Savo raised from the 18 000 (7.3%) at the beginning of 2012 to 21 600 (8.8%) at the beginning of 2018. About half of the prevalent T2D patients had admissions at KUH each year (Fig. 2). The mean (SD) age of the study cohort at the beginning of follow-up was 65.8 (13.2) years and 51.2% of them were men. About 55% of the study cohort had had at least one complication during 1996–2017. A total of 44 307 unique complication episodes in the hospital district of Northern Savo were extracted from the data. By restricting complications to diabetic ones with a start date between 2012–2016 to guarantee complete detailed cost data for the whole follow-up, the number of complication events included in the analyses was 16 148 for 7 895 patients with 102 803 person years of follow-up resulting in the crude total complication incidence of 15.7 per 100 person years. During 2012–2016 there were all the time around 13% of T2D patients who had at least one ongoing complication episode (Fig. 2). There were altogether 7 225 cardiovascular, 3 083 eye complication, 2 266 foot disorder, 2 222 cerebrovascular, 996 nephropathy, and 356 neurological episodes. Among these patients with complications, the mean (SD) age was 70.7 (11.3) years in the beginning of follow-up and 54.8% of them were men. In addition, at the time of a complication, 27.2% – 58.0% (59.4 – 90.7%) of these patients with incident (recurrent) complication had at least one recorded coexisting condition, on which congestive heart failure, any malignancy, and chronic pulmonary disease, were three most common coexisting conditions among patients with incident event, while for patients with recurrent event these three coexisting conditions were diabetes with chronic complications, congestive heart failure, and peripheral vascular disease (see Supplement tables S1 and S2 for details), respectively.

### Resource use and costs in patients with no history of T2D-related complications

The characteristics, the mean number of outpatient visits, and inpatient days by complication type of patients, as well as the mean one-year costs of those incident complications are presented in Table 1. Among patients with incident T2D-related complications, the patients with nephropathy complications were the oldest, whereas the patients with neurological complications were the youngest, on average, at the time of complication. The numbers of inpatient days were highest among patients with cerebrovascular complications and shortest in patients with eye complications. The highest mean one-year total costs were among patients with foot disorder complications.

**Table 1:** Characteristics, hospitalisation days and 1-year costs stratified by episode type among patients with no history of T2D-related complications. Complications are arranged in decreasing order of costs

Type of complication	n	Age (SD), years		Men (%)	Duration of T2D, mean (SD), years	Number of outpatient visits, mean (SD)	Number of inpatient days, mean (SD)	1-year costs (EUR), mean (SD)
Foot disorder	1 094	71.9 (11.4)	636 (58.1)		10.7 (7.6)	9.3 (17.5)	13.3 (21.4)	16 843 (25 237)
Nephropathy	371	72.5 (11.5)	236 (63.6)		11.3 (7.7)	10.6 (19.6)	14.7 (25.0)	15 264 (24 887)
Cardiovascular	1 870	72.0 (10.9)	1056 (56.5)		9.6 (7.2)	6.1 (12.6)	11.2 (18.5)	13 121 (19 121)
Cerebrovascular	1 367	72.1 (10.7)	699 (51.1)		9.7 (7.4)	6.0 (12.5)	14.8 (26.6)	12 744 (19 385)
Neurological	220	71.0 (12.1)	129 (58.6)		9.4 (7.0)	8.1 (13.4)	9.7 (17.6)	9 641 (17 971)
Eye complication	855	71.7 (13.1)	404 (47.3)		9.4 (7.1)	9.1 (12.1)	4.0 (8.9)	6 184 (10 568)

### Resource use and costs in patients with history of T2D-related complications

In patients with recurrent T2D-related complications (Table 2), the number of inpatient days were highest in patients with recurrent foot disorders complications and lowest in patients with recurrent eye complications. Among patients with recurrent T2D-related complications, the patients with cardiovascular complications were the oldest, whereas the patients with nephropathy complications were the youngest, on average. The highest mean one-year total costs were among patients with recurrent nephropathy complications.

**Table 2:** Characteristics, hospitalisation days and mean costs stratified by complication type (row) among patients with history of T2D-related complications. Complications are arranged in decreasing order of costs

Type of complication	n	Age (SD), years	Men (%)	Duration of T2D, mean (SD), years	Number of outpatient visits, mean (SD)	Number of inpatient days, mean (SD)	1-year costs (EUR), mean (SD)
Nephropathy	625	68.4 (10.3)	427 (68.3)	13.0 (7.0)	36.0 (49.1)	13.2 (19.8)	24 507 (32 342)
Foot disorder	1 172	72.9 (9.8)	806 (68.8)	12.2 (8.2)	12.2 (25.6)	13.7 (19.7)	17 666 (25 866)
Cardiovascular	5 355	75.4 (9.7)	3070 (57.3)	10.7 (7.5)	6.3 (12.1)	9.8 (15.5)	10 433 (18 828)
Neurological	136	71.7 (11.0)	75 (55.1)	13.1 (7.5)	11.4 (19.8)	8.8 (15.0)	10 296 (15 414)
Cerebrovascular	855	74.0 (10.1)	490 (57.3)	10.4 (7.5)	5.7 (12.2)	12.1 (22.8)	9 774 (13 194)
Eye complication	2 228	71.1 (11.7)	1163 (52.2)	15.2 (8.3)	10.5 (19.2)	4.0 (11.6)	6 304 (14 109)

### Distribution of complication costs according to hospital resource categories

**Fig. 3:**
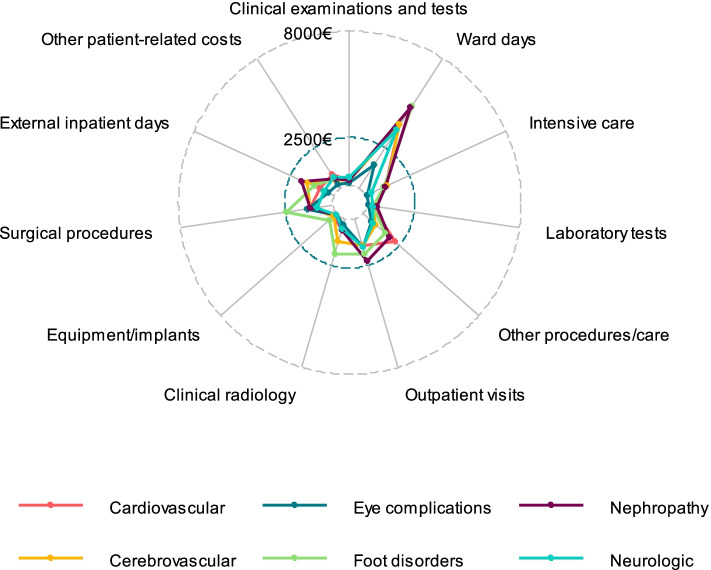
Distribution of complication costs according to hospital resource categories in patients without the history of T2D-related complications

**Fig. 4:**
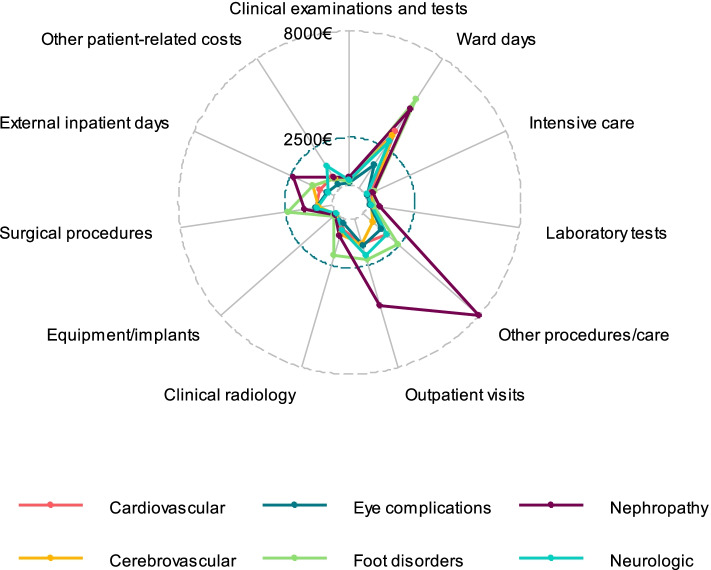
Distribution of complication costs according to hospital resource categories in patients with the history of T2D-related complications

Figures 3 and 4 show the distribution of complication costs according to hospital resource categories among patients with and without history of T2D-related complications, respectively. As shown in Fig. 3, inpatients days in hospital wards were a single resource category with highest costs in a case of all T2D-related complications in patients without history of T2D-related complications. However, among patients with history of T2D-related complication costs, the other procedure costs and outpatient visits were resource categories with highest costs in addition to inpatient days in hospital wards among patients with nephropathy-related complications (Fig. 4).

### Impact of T2D-related complications on subsequent second-year hospital costs

**Fig. 5:**
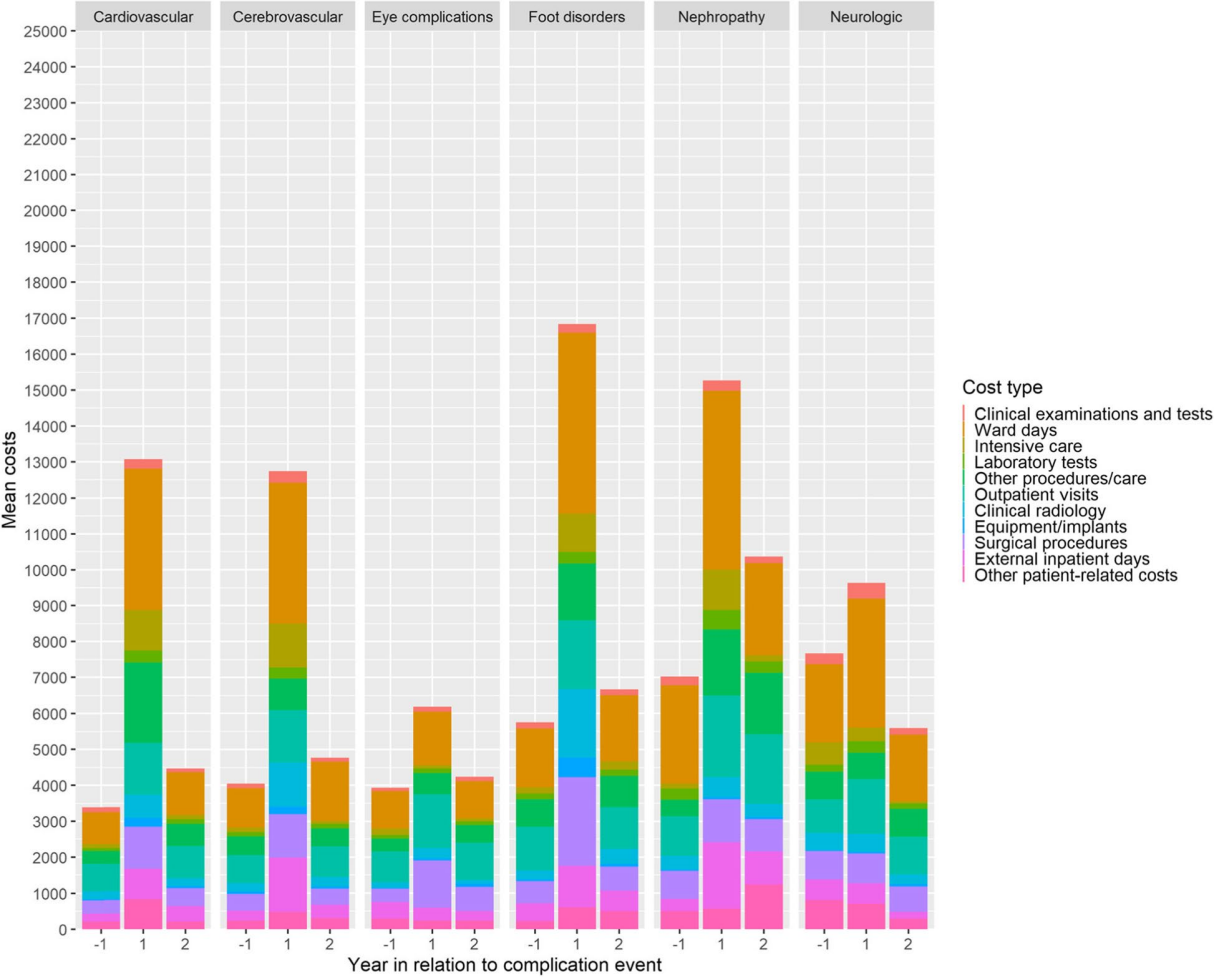
Mean total hospital treatment costs one year before and one and two years after of a complication event in patients without the history of T2D-related complications. See also Table 1

**Fig. 6:**
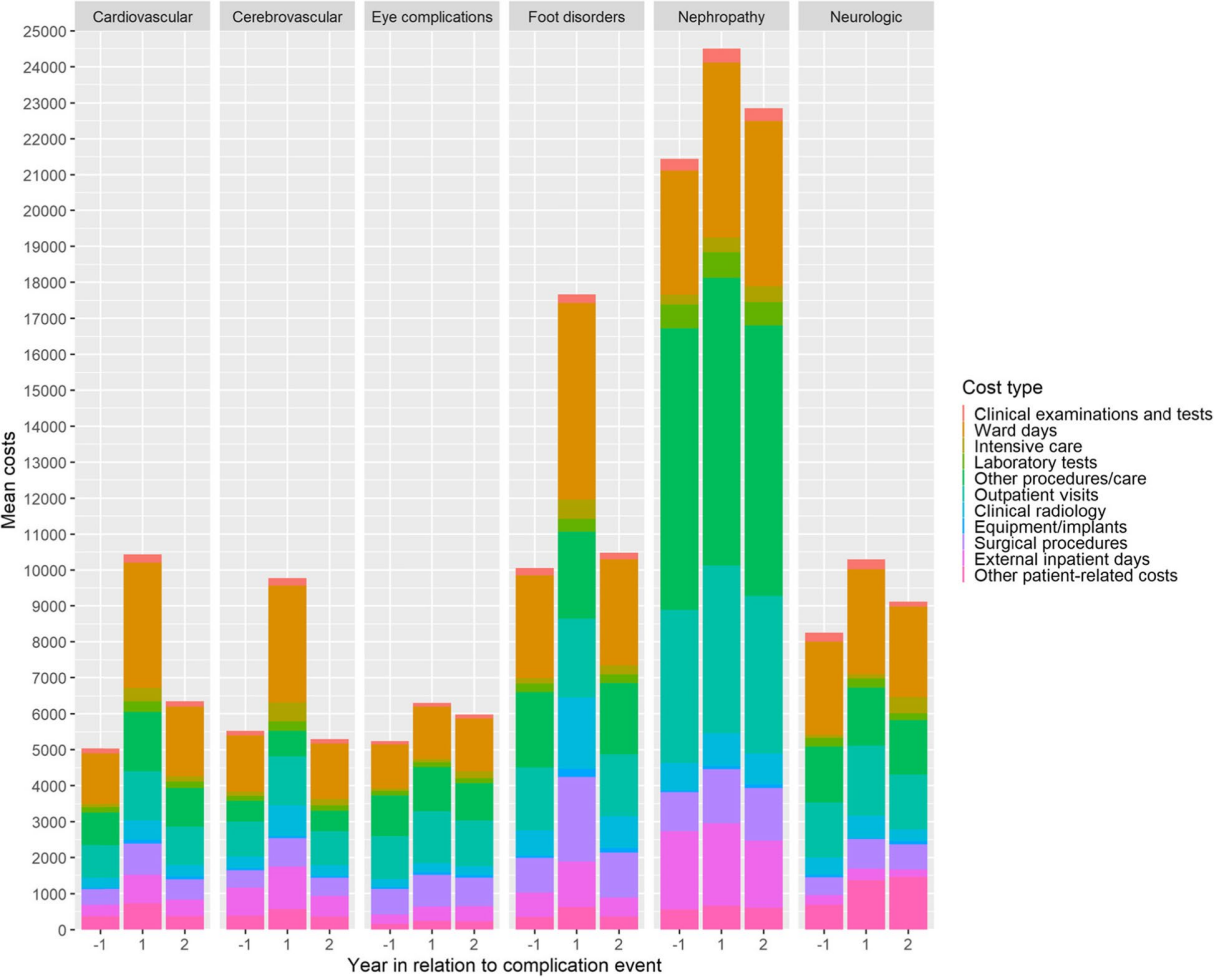
Mean total hospital treatment costs one year before and one and two years after of a complication event in patients with the history of T2D-related complications. See also Table 2

When considering the mean total hospital treatment costs in patients with incident T2D-related complication one year before and after of their index complications (Fig. 5), the mean total hospital treatment costs were higher during the second year after the index complications in all complication types with exception of neurological complications in comparison to the year before the complication. Among recurrent complications (Fig. 6), costs were particularly high among nephropathic complications. For all other recurrent complications than cerebrovascular ones the second-year costs were clearly higher than costs of one year before the complication.

### Sociodemographic and clinical determinants of T2D-related complication costs

Tables 3 and 4 show the results of regression models for incident and recurrent complication costs, respectively. As shown in Table 3, in patients with incident complications, age has a slight, but significant decreasing effect on one-year costs (excluding the neurologic complications). In addition, death during the period of one year after the complication event had a significant increasing effect on adjusted one-year costs (except for foot disorders). In patients with incident (recurrent) complication, moderate or severe renal disease, which was diagnosed on average 7.2% (14.8%) of patients as a coexisting condition, doubled or even tripled the one-year treatment costs, when dealing with cardiovascular, foot disorders or eye complications.

**Table 3:** Results of multivariable log-gamma regression models for the one-year costs of incident T2D-related complications

	Cerebrovascular	Cardiovascular	Nephropathy	Foot disorders	Eye complications	Neurologic
n	1367	1870	371	1094	855	220
Intercept (€)^a^	9663.62 **	11,143.52 **	7517.73 **	13,492.7 **	4252.5 **	4785.58 **
Sex (woman)^b^	0.85 *	0.83 **	0.98	0.90	0.93	1.25
Patient's age at the time of hospitalization (centered at 75 years of age, coefficient for 10 years increase)	0.81 **	0.85 **	0.59 **	0.88 **	0.88 **	0.86
Duration of T2D at the time of hospitalization (coefficient for 5 years increase)	1.05 *	1.02	1.06	1.02	1.02	1.00
Death during the period of one year since the complication event	1.53 **	1.41 **	1.49 *	1.16	2.21 **	2.03 *
Rheumatological disease	1.28	1.74 **	1.08	1.69 *	2.88 **	1.62
Dementia	0.49 **	0.52 **	1.27	0.70	0.88	0.31 *
Cerebrovascular disease	1.07	1.08	0.53	0.97	1.19	2.31 *
Congestive Heart Failure	1.36 *	1.55	1.47 *	1.30 *	1.29	1.26
Moderate or severe renal disease	1.33	2.25 **	1.03	1.75 **	3.21 **	1.95
Chronic Pulmonary Disease	1.00	1.07	1.09	1.28	1.18	0.98
Myocardian infarction	1.39	NA	0.93	1.01	0.92	1.87
Any malignancy	1.11	1.07	1.30	1.34	1.98 **	2.00 *
Peripheral vascular disease	1.36	1.36 *	1.69 *	0.89	1.97 *	1.67
Peptic ulcer disease	1.45	1.06	1.07	1.02	1.68	0.16
Chronic liver disease	1.27	1.22	1.08	1.35	1.15	1.96
McFadden’s pseudo-*R*^2^	0.107	0.090	0.156	0.065	0.165	0.248

Regression coefficients (except the intercept) are exponentiated so that the interpretation for the coefficient is relative to the reference value, i.e. coefficient tells how many times larger (or smaller) the one-year costs are in the current group (on condition that other factors remain the same). For example, the predicted one-year cost for men with cerebrovascular complication (and with all factors in reference values) is 9663.62€ while the one-year cost for women is 0.85*9663.62 = 8214.077€

*NA* Not Applicable

*P*-values: ** *p* < 0.01, * *p* < 0.05

^a^ Corresponds with the costs of 75-year-old men with newly diagnosed T2D, no coexisting comorbidities and who will stay alive a period of one year since the complication event

^b^ A relative difference in treatment costs between men and women

**Table 4:** Results of multivariable log-gamma regression models for one-year costs of recurrent T2D-related complications

	Cerebrovascular	Cardiovascular	Nephropathy	Foot disorders	Eye complications	Neurologic
n	855	5355	625	1172	2228	136
Intercept (€)^a^	8980.94 **	7865.37 **	4723.3 **	12,005.25 **	3208.07 **	5997.52 **
Sex (woman)^b^	0.91	0.90 *	1.20	0.96	1.10	0.87
Patient's age at the time of hospitalization (centered at 75 years of age, coefficient for 10 years increase)	0.86 **	0.83 **	0.77 **	0.82 **	0.94	0.89
Duration of T2D at the time of hospitalization (coefficient for 5 years increase)	1.00	1.01	1.07	1.03	1.00	1.00
Death during the period of one year since the complication event	1.05	1.28 **	1.20	1.40 **	1.87 **	2.12 **
Number of earlier complication episodes	0.97	1.00	1.03	1.05 *	1.04 **	0.87 **
Exceptionally high earlier costs	1.62	2.19 **	2.39 **	1.75 **	3.57 **	4.61 **
Rheumatological disease	1.77	1.73 **	1.11	1.33 *	1.54 **	9.87 **
Dementia	0.84	0.87	0.36 **	0.69 **	0.72 *	1.56
Cerebrovascular disease	0.80 *	1.06	0.89	0.85	1.38 *	1.06
Congestive Heart Failure	0.98	0.98	1.10	0.88	1.15	0.91
Diabetes with chronic complications	1.50 **	1.32 **	1.68 **	1.24 **	0.94	1.70
Moderate or severe renal disease	1.66 **	1.49 **	1.55 **	2.00 **	3.20 **	0.97
Chronic Pulmonary Disease	1.29	1.23 **	1.25	1.17	1.09	0.78
Myocardian infarction	1.00	1.11	1.32*	1.26 *	1.51 **	1.30
Any malignancy	1.03	1.37 **	1.12	0.93	1.56 **	2.21 **
Peripheral vascular disease	1.29*	1.26 **	1.28	0.78 **	1.73 **	1.42
Peptic ulcer disease	1.36	1.43 **	2.19 **	1.04	1.25	0.24 **
Chronic liver disease	2.03	1.48 *	1.35	1.01	1.18	3.46
McFadden’s pseudo-*R*^2^	0.094	0.115	0.267	0.146	0.230	0.327

Regression coefficients (except the intercept) are exponentiated so that the interpretation for the coefficient is relative to the reference value, i.e. coefficient tells how many times larger (or smaller) the one-year costs are in the current group (on condition that other factors remain the same). For example, the predicted one-year cost for men with cerebrovascular complication (and with all factors in reference values) is 8980.94€ while the one-year cost for women is 0.91*8980.94 = 8172.655€

*P*-values: ** *p* < 0.01, * *p* < 0.05

^a^ Corresponds with the costs of 75-year-old men with newly diagnosed T2D, no coexisting comorbidities and who will stay alive a period of one year since the complication event

^b^ A relative difference in treatment costs between men and women

Among the patients with recurrent complications, age had also significant decreasing effect on one-year costs (except for eye and neurologic complications). A diagnosis code “diabetes with chronic complications”, which was recorded for 13.3%—67.6% of patients (depending on the type of a recurrent event), had also significant increasing effect on one-year costs (except for eye complications). Furthermore, moderate or severe renal disease as a coexisting condition increased one-year costs significantly in patients with recurrent events (except for neurologic complications). The average marginal effects for the regression coefficients, i.e. the average costs in euros in regard to unit increase of a variable, of incident and recurrent models are presented in Supplement tables S4 and S5.

## Discussion

The present study showed the substantial hospital treatment costs of T2D-related complications as average per-patient first-year treatment costs varied from 6 184 to 24 507 euros with total raw mean costs of 11 708 euros. On average, the treatment costs of incident complications were higher than the treatment costs of recurrent events (except for recurrent nephropathy complications). In case of all considered complications, a resource category with highest category-specific costs was the number of treatment days in hospital wards. Whereas, in a case of nephropathy complications, “other procedures” category (i.e., driven by haemodialysis in patients with an end-stage renal disease) had the highest category-specific costs in patients with the recurrent complications. In addition, it is notable that the patients with recurrent nephropathy complications were also younger than patients with other complications, on average. This finding is a line with a recent real-world data study indicating patients with nephropathy complications as a patient subgroup with highest treatment costs [14]. Although many of these T2D patients with nephropathy complications die before ending-up to actual end-stage renal disease [15], this finding together with the observed average duration of T2D among these patients indicate the early onset of type 2 diabetes and potential challenges with the proper management of hyperglycaemia.

The results of our study also showed that the mean complication costs were higher (with some exceptions) during subsequent second-year after the year of event than during a year before an index year of event indicating potential increased long-term service need due to occurred T2D-related complications. Furthermore, our multivariable analyses showed that many of coexisting conditions at the time of a complication event account significantly for one-year cost variability. As showed above, over 30% (60%) of patients with an incident (recurrent) complications had at least one coexisting condition at the time of a complication event indicating the importance of these associations in the accumulation of treatment costs. This finding is in a line with previous studies showing the type and combination of comorbidities may impact on access, intensity, and health care utilization patterns of care [16–19]. Our study also showed that very high one-year complication costs (i.e., ≥ 50 000 euros) during an initial index complication are a significant predictor of high costs also in a case of recurrent hospitalisation in all types of studied complications. Overall, the cost estimates of our study agree with the results of previous studies reporting the impact of T2D-related complications on healthcare costs [8, 9].

In addition, in the era of the “precision medicine”, subgroup-specific cost estimates are increasingly needed (and required by HTA bodies) e.g., in health economic modelling studies evaluating the cost-effectiveness of new therapies for T2D in more and more specific patient subgroups. Our results and the accompanied web-application (https://uef-phoru.shinyapps.io/T2DCost/) allow to “predict” the detailed costs by a selection the patient characteristics, which hopefully provides some useful insights for end-users, when they consider trade-offs between accuracy and usability of these estimates e.g., when parametrising a health economic model under development, or just to increase awareness and understanding of the applicability as well as the possibilities (and limits) of health economic modelling of real-world data. In any case, it must be recognized that the model (and the online tool) only describes the systematic patterns that can be observed from the real-world data – those are not suggestions how things should go, and all the predictions must be carefully assessed before making any strong conclusions.

Our study has strengths. One of the advantages of our study is that we had access to very detailed billing data that could be linked to nationwide data to guarantee complete follow-up. We also applied the multivariable regression analysis to study the sociodemographic and clinical (i.e., the duration of disease and coexisting conditions) determinants of T2D-related complications with a developed website application tool providing an opportunity to study the effect of selected baseline sociodemographic variables and co-existing comorbidities on the predicted complication costs in patients with and without the history of T2D-related complications at baseline. As far as we know, there is no similar type of real-world data studies that provide a such detailed description of costs in patients with T2D-related incident or recurrent complications. However, there are naturally also limitations that need to be considered when interpreting the results of the present study. First, we applied a real-world dataset collected mainly from the electronic patient information systems of one university hospital. This potentially limits the generalisation of study results since there might be differences in treatment practices and the unit costs of resources across hospitals. Second, we were not able to consider all cost components, such as travel costs, relevant for decision-making and resource allocation in health care. In some cases, such as in a case of nephropathy complications, this may underestimate the total costs of care. For example, it has been previously estimated that travel costs account for around 15% of total treatment costs for haemodialysis [20]. More recently, also the Finnish Social Insurance Institution has reported high travel costs for patients undergoing haemodialysis being on average 10,000€ per patients and even exceeding 60,000€ for some patients [21]. Third, we were not able to consider potential increase in the use of primary care services associated with studied complications. However, acute T2D-related complications are mainly treated and monitored at hospitals in Finland, so the obtained estimates probably do not underestimate the total treatment costs of complications significantly. Fourth, the use of one-year costs left room for the potential overlapping of different types of complication episodes within that period. Due to the above limitations of the current study, a further real-world study linking individual-level data on health services utilization retrieved from nationally presentative data sources, separating the types of complication combinations [22], and including all relevant cost components, such as T2D-related productivity costs [23], could provide even more holistic insight in the costs of T2D-related complications in Finland.

## Conclusions

As a summary, this study shows updated Finnish one-year cost estimates for T2D-related complications treated in the university hospital setting. In a case of all complications (except for recurrent nephropathic complications), the number of treatment days in hospital wards is the most significant cost driver. The results of our study highlight the role of guideline implementation, effective preventive treatments for T2D, as well as the importance of treatment adherence to avoid these costly complications.

## Supplementary Information

**Additional file 1.** 

## Data Availability

The datasets analysed during this study were used under licences for current study and so are not publicly available. Data are however available from the corresponding author on reasonable request and with permissions from the FinDM project and the Finnish Social and Health Data Permit Authority Findata. The cost calculator based on the data is freely available at: https://uef-phoru.shinyapps.io/T2DCost/.

## Abbreviations

CI: Confidence interval
CRHC: Care Register for Health Care
KUH: Kuopio University Hospital
SD: Standard deviation
T2D: Type 2 Diabetes
