# Supplementary material for: Hospital treatment costs associated with incident complications in patients with type 2 diabetes—real-world study based on electronic patient information systems
Source: BMC Health Serv Res. 2022 Apr 9;22:469. doi: 10.1186/s12913-022-07895-6 (PMC8994912; doi:10.1186/s12913-022-07895-6)
Supplement: Supplementary file 1 — Additional file 1. [file 12913_2022_7895_MOESM1_ESM.docx]

**Supplementary Information**

Cost calculator freely available at: <https://uef-phoru.shinyapps.io/T2DCost/>

Supplement table S1. –Number (%) of different coexisting comorbities and other dichotomous covariates in patients with incident complications

|  | Cerebrovascular | Cardiovascular | Nephropathy | Foot disorders | Eye complications | Neurologic |
| --- | --- | --- | --- | --- | --- | --- |
| Women | 668 (48.87%) | 814 (43.53%) | 135 (36.39%) | 458 (41.86%) | 451 (52.75%) | 91 (41.36%) |
| Death | 212 (15.51%) | 275 (14.71%) | 95 (25.61%) | 150 (13.71%) | 47 (5.5%) | 21 (9.55%) |
| Rheumatological disease | 34 (2.49%) | 41 (2.19%) | 14 (3.77%) | 47 (4.3%) | 22 (2.57%) | 3 (1.36%) |
| Dementia | 83 (6.07%) | 65 (3.48%) | 18 (4.85%) | 47 (4.3%) | 35 (4.09%) | 13 (5.91%) |
| Cerebrovascular disease | 31 (2.27%) | 98 (5.24%) | 20 (5.39%) | 113 (10.33%) | 51 (5.96%) | 23 (10.45%) |
| Congestive Heart Failure | 114 (8.34%) | 16 (0.86%) | 104 (28.03%) | 163 (14.9%) | 64 (7.49%) | 21 (9.55%) |
| Chronic Pulmonary Disease | 93 (6.8%) | 87 (4.65%) | 41 (11.05%) | 86 (7.86%) | 49 (5.73%) | 15 (6.82%) |
| Moderate or severe renal disease | 40 (2.93%) | 43 (2.3%) | 18 (4.85%) | 61 (5.58%) | 24 (2.81%) | 9 (4.09%) |
| Myocardian infarction | 68 (4.97%) | 0 (0%) | 42 (11.32%) | 96 (8.78%) | 49 (5.73%) | 18 (8.18%) |
| Any malignancy | 100 (7.32%) | 142 (7.59%) | 53 (14.29%) | 85 (7.77%) | 54 (6.32%) | 22 (10%) |
| Peripheral vascular disease | 74 (5.41%) | 102 (5.45%) | 41 (11.05%) | 32 (2.93%) | 28 (3.27%) | 17 (7.73%) |
| Peptic ulcer disease | 17 (1.24%) | 24 (1.28%) | 7 (1.89%) | 17 (1.55%) | 10 (1.17%) | 2 (0.91%) |
| Chronic liver disease | 12 (0.88%) | 12 (0.64%) | 9 (2.43%) | 12 (1.1%) | 10 (1.17%) | 3 (1.36%) |

Supplement table S2. –Number (%) of different coexisting comorbities and other dichotomous covariates in patients with recurrent complications

|  | Cerebrovascular | Cardiovascular | Nephropathy | Foot disorders | Eye complications | Neurologic |
| --- | --- | --- | --- | --- | --- | --- |
| Women | 365 (42.69%) | 2285 (42.67%) | 198 (31.68%) | 366 (31.23%) | 1065 (47.8%) | 61 (44.85%) |
| Death | 150 (17.54%) | 898 (16.77%) | 104 (16.64%) | 221 (18.86%) | 123 (5.52%) | 10 (7.35%) |
| Exceptionally high earlier costs | 7 (0.82%) | 79 (1.48%) | 96 (15.36%) | 61 (5.2%) | 30 (1.35%) | 3 (2.21%) |
| Rheumatological disease | 29 (3.39%) | 170 (3.17%) | 17 (2.72%) | 66 (5.63%) | 49 (2.2%) | 2 (1.47%) |
| Dementia | 42 (4.91%) | 294 (5.49%) | 10 (1.6%) | 60 (5.12%) | 85 (3.82%) | 9 (6.62%) |
| Cerebrovascular disease | 337 (39.42%) | 468 (8.74%) | 54 (8.64%) | 129 (11.01%) | 176 (7.9%) | 15 (11.03%) |
| Congestive Heart Failure | 122 (14.27%) | 1360 (25.4%) | 168 (26.88%) | 246 (20.99%) | 250 (11.22%) | 19 (13.97%) |
| Diabetes with chronic complications | 114 (13.33%) | 820 (15.31%) | 365 (58.4%) | 430 (36.69%) | 1506 (67.59%) | 61 (44.85%) |
| Moderate or severe renal disease | 27 (3.16%) | 263 (4.91%) | 365 (58.4%) | 102 (8.7%) | 140 (6.28%) | 10 (7.35%) |
| Chronic Pulmonary Disease | 60 (7.02%) | 517 (9.65%) | 53 (8.48%) | 122 (10.41%) | 87 (3.9%) | 18 (13.24%) |
| Myocardian infarction | 82 (9.59%) | 843 (15.74%) | 82 (13.12%) | 190 (16.21%) | 138 (6.19%) | 11 (8.09%) |
| Any malignancy | 67 (7.84%) | 427 (7.97%) | 55 (8.8%) | 88 (7.51%) | 124 (5.57%) | 6 (4.41%) |
| Peripheral vascular disease | 94 (10.99%) | 606 (11.32%) | 99 (15.84%) | 600 (51.19%) | 185 (8.3%) | 17 (12.5%) |
| Peptic ulcer disease | 16 (1.87%) | 76 (1.42%) | 21 (3.36%) | 31 (2.65%) | 20 (0.9%) | 3 (2.21%) |
| Chronic liver disease | 5 (0.58%) | 38 (0.71%) | 24 (3.84%) | 14 (1.19%) | 14 (0.63%) | 2 (1.47%) |

Supplementary Table S3 – Diagnosis and procedure codes used to define major complications of type 2 diabetes (source: <http://urn.fi/URN:ISBN:978-952-343-492-9> , pages 14 – 15, Table 2)


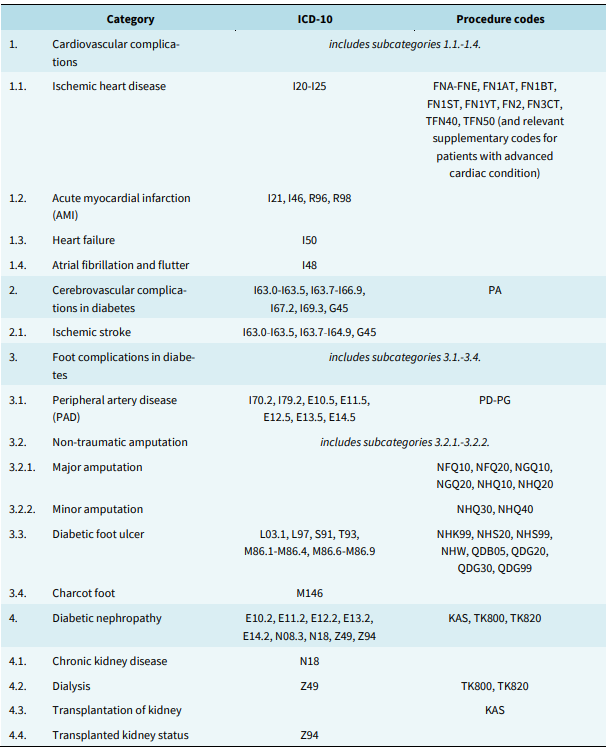

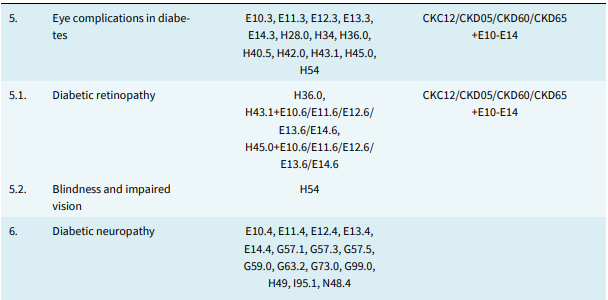


Supplement table S4. –Average marginal effects for incident related T2D-related complications’ log-gamma regression models. Average change in euros (€) in relation to reference category or increase of stated years for continuous variables on condition that other variables remain in the same values.

|  | Cerebrovascular | Cardiovascular | Nephropathy | Foot disorders | Eye complications | Neurologic |
| --- | --- | --- | --- | --- | --- | --- |
| Sex (woman) | -2725.44 | -2108.25 | -8377.93 | -2071.73 | -829.80 | -1558.99 |
| Patient's age at the time of hospitalization (centered at 75 years of age, coefficient for 10 years increase) | 1302.14 | 895.52 | 4098.88 | 4967.70 | 4458.70 | 6908.13 |
| Duration of T2D at the time of hospitalization (coefficient for 5 years increase) | 836.67 | 983.04 | -9880.06 | -535.78 | 1137.80 | 8355.75 |
| Death during the period of one year since the complication event | 3066.55 | 2610.43 | 1212.00 | 5007.73 | 924.17 | 6705.97 |
| Rheumatological disease | -22.17 | 844.61 | 1422.77 | 4104.29 | 1099.95 | -172.45 |
| Dementia | 3929.86 | 5732.69 | 6019.55 | 4420.96 | 1669.87 | 2296.02 |
| Cerebrovascular disease | 5366.94 | 4468.00 | 6251.01 | 2523.85 | 5175.64 | 7069.51 |
| Congestive Heart Failure | -8937.24 | -8633.38 | 3819.99 | -5924.82 | -821.40 | -11661.74 |
| Moderate or severe renal disease | 653.58 | 294.40 | 892.75 | 282.01 | 109.02 | 43.31 |
| Chronic Pulmonary Disease | 3619.70 | 10586.06 | 482.40 | 9427.39 | 7582.52 | 6699.78 |
| Myocardian infarction | 4204.35 | 0.00 | -1086.48 | 211.47 | -509.37 | 6246.75 |
| Any malignancy | 4692.99 | 732.47 | 1040.94 | 289.09 | 3372.67 | -18392.20 |
| Peripheral vascular disease | 3886.74 | 4035.9 | 8238.30 | -1868.11 | 4409.25 | 5102.45 |
| Peptic ulcer disease | 3100.25 | 7270.58 | 1182.19 | 8779.54 | 6876.87 | 4797.78 |
| Chronic liver disease | -1979.26 | -2414.74 | -364 | -1796.21 | -449.79 | 2297.56 |

Supplement table S5. –Average marginal effects for recurrent related T2D-related complications’ log-gamma regression models. Average change in euros (€) in relation to reference category or increase of stated years for continuous variables on condition that other variables remain in the same values.

|  | Cerebrovascular | Cardiovascular | Nephropathy | Foot disorders | Eye complications | Neurologic |
| --- | --- | --- | --- | --- | --- | --- |
| Sex (woman) | -1427.25 | -1972.30 | -6699.49 | -3489.22 | -454.12 | -1268.55 |
| Patient's age at the time of hospitalization (centered at 75 years of age, coefficient for 10 years increase) | 329.42 | 3243.58 | 2959.79 | -1360.12 | 3059.95 | 8475.03 |
| Duration of T2D at the time of hospitalization (coefficient for 5 years increase) | -2116.67 | 612.43 | -2920.34 | -2873.09 | 2221.19 | 598.93 |
| Death during the period of one year since the complication event | 6881.36 | 4079.97 | 7678.45 | 176.48 | 1114.35 | 13277.87 |
| Number of earlier complication episodes | 2455.77 | 2125.93 | 5714.23 | 2822.50 | 613.66 | -2713.22 |
| Exceptionally high earlier costs | -180.14 | -167.74 | 2438.34 | -2194.56 | 980.85 | -963.93 |
| Rheumatological disease | 512.31 | 2608.14 | 4699.15 | 5895.85 | 4305.07 | 8049.33 |
| Dementia | -1690.19 | -1492.86 | -25958.22 | -6581.55 | -2294.56 | 4758.62 |
| Cerebrovascular disease | 3971.24 | 2871.66 | 13229.08 | 3797.35 | -452.41 | 5686.58 |
| Congestive Heart Failure | 16.34 | 116.24 | 1715.16 | 436.81 | -24.05 | 7.17 |
| Diabetes with chronic complications | 4684.95 | 8157.57 | 22115.58 | 9865.17 | 8761.73 | 16368.00 |
| Moderate or severe renal disease | 4927.91 | 4174.74 | 11079.07 | 12235.55 | 8008.39 | -316.78 |
| Chronic Pulmonary Disease | -31.75 | 1132.09 | 7057.74 | 4016.07 | 2835.31 | 2848.17 |
| Myocardian infarction | -261.10 | -8.11 | 739.29 | 856.28 | 271.08 | -1478.99 |
| Any malignancy | 2979.05 | 3728.82 | 19885.70 | 753.21 | 1539.79 | -15313.53 |
| Peripheral vascular disease | 2507.12 | 2390.44 | 6356.42 | -4393.96 | 3771.71 | 3737.73 |
| Peptic ulcer disease | 5584.59 | 5725.55 | 2601.50 | 5035.09 | 2980.34 | 24508.10 |
| Chronic liver disease | -921.64 | -1079.38 | 4755.88 | -749.62 | 655.37 | -1540.80 |
